# A universal home-visit programme to tailor support to first-time parents: a qualitative case study on parents’ perspectives

**DOI:** 10.1186/s12889-025-24114-z

**Published:** 2025-09-09

**Authors:** Ann-Christine Andersson, Marie Golsäter

**Affiliations:** 1 Jönköping Academy for Improvement of Health and Welfare, School of Health and Welfare, Jönköping University, Jönköping, Sweden; 2 The Child Health Care Service, Region Jönköping County, Jönköping, Sweden; 3 CHILD Research Group, School of Health and Welfare, Jönköping University, Jönköping, Sweden; 4 Futurum – Academy for Health and Care, Region Jönköping County, Jönköping, Sweden

**Keywords:** Child health services, Extended home-visits, First-time parents, Reflexive thematic analysis

## Abstract

**Background:**

The first year of a child’s life is essential for promoting a healthy life, and the transition to becoming a parent can be a challenge; parents need to develop confidence in their own capacity to care for their child. The national Child Health Services programme in Sweden offers parental support, both on a universal level and in accordance with the individual family’s needs. This study explores parents’ experiences of an extended home-visit programme offered through a Family Centre to all first-time parents in a municipality.

**Methods:**

This case study is based on a qualitative reflexive thematic approach, using interviews with first-time parents. Fourteen mothers and five fathers who had taken part in the extended home-visit programme were interviewed by telephone between February 2023 and April 2024.

**Results:**

The analysis resulted in one overarching theme, Universal home-visits create preconditions for tailored parental support, and four subthemes: Relations and continuity are essential; Different professional competencies complement each other and reinforce support; Home environments increase the feeling of security; and The universal approach facilitates acceptance. The parents had mainly positive experiences and thought that extended home-visits could benefit all first-time parents, not only those in socioeconomically vulnerable areas.

**Conclusions:**

This study shows that universal extended home-visits create preconditions for more family-tailored support and strengthen first-time parents in developing their parenthood, which in turn increases the possibilities for optimal growth and development of the child.

**Supplementary Information:**

The online version contains supplementary material available at 10.1186/s12889-025-24114-z.

## Background

The first year of a child’s life is essential for promoting a healthy life [1]. To reduce toxic stress and promote foundational social-emotional health, a nurturing and responsive parent–child relationship is essential [2]. The World Health Organization’s (WHO) initiative on nurturing care to promote a safe childhood for every kid is based on care, protection, prevention, and support for children early in life [3].

For optimal growth and development of each child, both children’s and parents’ well-being are central [4]. Becoming a parent means transitioning from one life situation to another, a process during which the parents develop confidence in their own capacity to care for their child [5, 6].

Factors that can affect parenting and cause stress are, for example, mental health problems and a lack of access to support [7]. In the transition to parenthood, parents need support, for example by discussing their parenting and receiving individual support, firstly from relatives and friends but also from health care professionals such as those at the Child Health Services (CHS) [8].

The National Strategy for Strengthened Parental Support in Sweden [9] highlights the importance of early parental support and further describes how early support can promote health in both parents and children. Within the frame of the national CHS programme in Sweden, parental support is offered both on a universal level and on a targeted level based on the individual family’s needs [10, 11].

The CHS in Sweden emphasizes proportional universalism [12] and provides free well-child care for children aged 0–5 years and their parents. The CHS goal is to promote the child’s health and development based on a national health monitoring programme. This includes measuring the child’s growth, following the child’s development, and promoting a healthy life style. The health monitoring programme comprising 16 age-specific health visits where the family is supposed to meet the same nurse through the programme. In each visit, the CHS nurse is supposed to consider each family situation in a caring dialogue tailored to the unique needs of the family and also offer additional visits when needed [10]. Four of the visits is conducted in collaboration with a general practitioner as team visits, and when the child is a newborn and at the age of eight months, the health visits are home-visits [11]. The home-visits purpose is to facilitate the creation of a caring relationship between the CHS nurse and the parents as a foundation to promote the child’s health and development.

A scoping review by Welsh et al. [13] showed that supporting parents at home is helpful for positive parenting behaviour both on a universal level and for disadvantaged groups. Further, Staal et al. [14] show that home-visits provide a better opportunity to identify families in need of extended intervention from CHS than visits carried out at a child health clinic.

The purpose of offering families more universal home-visits is to create a better opportunity to identify risk and protective factors in the family in partnership with the parents and thereby provide earlier support and promote the child’s health and development [15]. In some Swedish regions, extended home-visits by CHS nurses and social workers have been introduced for first-time parents based on the results from the Rinkeby programme [16], mainly in more socioeconomically vulnerable areas [see e.g. [17–19].

Parents in Bäckström et al. [20] described how receiving parental support in their home, as a safe place, was helpful and strengthened their parenting role. Experiences of receiving more tailored support and strengthening their relationship with the nurse through an extended home-visit programme are also described by Norwegian fathers [21].

Healthcare professionals who work with extended home-visit programmes also describe how the home-visits facilitate the provision of parental support. The dialogues during the home-visits focus more clearly on what the parents need to discuss. In addition, collaboration between the different professionals strengthens the opportunities for more person-centred parenting support [22]. In the context of paediatric populations, the concept of family-centred care is used [23], where care is planned around the whole family, thus recognizing all family members and not only the child [24]. One way to organise CHS units is as a part of a Family Centre, including antenatal and midwifery services, social workers and an open preschool. The collaboration between the different professionals within the Family Centre is supposed to strengthen parental support further [10]. Family-centred care can be an important key to facilitating support to first-time parents on their terms [25], as illustrated by the microsystem theory [26].

### Microsystem theory

**Fig. 1:**
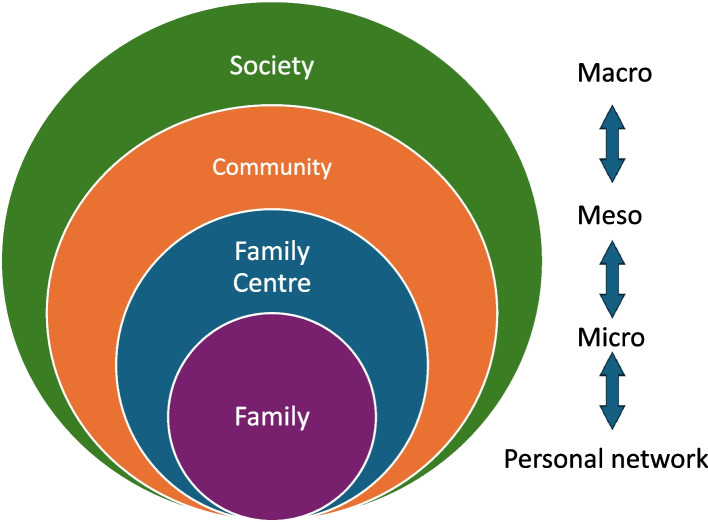
Microsystem theory applied to CHS

Healthcare is an interdependent system containing many different layers. Microsystem theory can be used as a lens to illustrate different perspectives and how they are interconnected and interdependent [26]. In microsystem theory, the needs of the patient and their personal network are always in focus. Applied to the CHS, the family is at the centre (personal network perspective), and the Family Centre constitutes the microsystem, surrounded by the community (meso perspective) and society as a whole (macro perspective) (Fig. 1). In this theory, the microsystem is the core of healthcare delivery [26], where value is created – in this case the Family Centre. Therefore, it is important that the different professions, such as CHS nurses and social workers, work together as a unit, even when they belong to different organisations. The Region in which the Family Centre is located, and this study is conducted, has applied ‘quality as strategy,’ a long-standing application of QI and microsystem theory [27].

A well-functioning microsystem increases quality and safety [28]. To accomplish the purpose of the extended home-visit programme, the Family Centre needs to form its microsystems together with the family. Person-centredness implies coproduction together with the whole family – a family-centred care approach [23] – to create an improved service [29].

In 2017, the Swedish state and the health care regions established the Child Health Service Accessibility Agreement [30]. The goal was to increase the accessibility of CHS services, especially in socioeconomically vulnerable areas. Some Swedish regions have since then started to carry out extended home-visit programmes where CHS nurses and social workers together visit first-time parents to expand tailored parental support based on the individual family’s situation [20, 22, 31]. Extended home-visit programmes conducted by CHS nurses and social workers contribute to safeguarding first-time parents [16] and building trustful relations towards Swedish authorities such as the CHS and social services [16, 32].

When the positive results from the Rinkeby programme [16] were spread, an extended home-visit programme was introduced in more socioeconomically challenging areas in larger cities. The question was raised of how to introduce a similar programme in smaller municipalities where areas of socioeconomic vulnerability were more scattered. An essential concern when introducing such a programme in smaller municipalities is to reduce the risk of parents feeling singled out or stigmatized when they are offered the programme. To address this concern, one municipality and region cooperating within the frame of a Family Centre decided to start introducing a universal home-visit programme. The programme was initiated in the municipality due to both their socioeconomical conditions and the willingness to try the programme. The programme involved four joint home-visits by the Family Centre’s CHS nurse and social worker. The programme was offered to all parents having their first child in the municipality rather than just to families in more socioeconomically challenging situations. To further explore how to tailor an extended home-visit programme to parents who have access to a Family Centre and live in municipalities outside large city areas, it is necessary to explore parents’ acceptance and experiences of the programme when it is introduced to all first-time parents. Therefore, the aim of this study is to explore parents’ experiences of an extended home-visit programme offered through a Family Centre to all first-time parents in a municipality.

## Methods

### Design

This is a qualitative reflexive case study with a thematic approach [33, 34], based on interviews conducted between February 2023 and April 2024. The research is reported in accordance with the Reflexive Thematic Analysis Reporting Guidelines (RTARG) [35, 36].

### Settings

The study was performed in a municipality in Region Jönköping County with a population of around 31,900 inhabitants in an area of about 990 square kilometres. This municipality was the first in the region to start an extended home-visit programme. At the Family Centre in question, nine CHS nurses and three social workers were employed. The programme was offered to all first-time parents in the municipality. The families were also invited to use other facilities at the Family Centre, such as the open pre-school. The antenatal and midwifery service are also located in the same place, which may facilitate collaboration between the professionals supporting the families.

### The home-visit programme

In 2019, the Region initiated a project based on the Rinkeby programme [18, 37]. An extended home-visit programme was created, including four home-visits to all first-time parents at a pilot Family Centre. The extended home-visits took place when the child was newborn, four months, eight months, and fifteen months old. Compared to the national CHS programme, this health visit programme added two more home-visits, one of which (at the age of 15 months) was an additional visit and one was an exchange from a clinic visit (at the age of 4 months). The nurse who encountered the family at the clinic conducted the home-visits, and it was also the same nurse and social worker that attended all the home-visits with the family. The families could also have further contact with the social worker at the Family Centre. The choice not to start with six visits, as in the Rinkeby programme, was based on the available resources and the Region's strategies for quality improvement when introducing new methods [27, 38]. Managers from the CHS and social service organization, with the support of the CHS management, collaborated together with the involved CHS nurses and social workers to introduce the programme, further described by Golsäter and Andersson [25].

### Participants

Parents who had participated in the extended home-visit programme at the Family Centre were asked to be interviewed about their experiences. The CHS nurse asked them, and if they wanted to be interviewed, they left a phone number and were then contacted by the researchers to book an appointment. Twenty-two parents left their phone numbers. One parent did not answer when contacted by the researcher, and three others (two fathers and one mother) declined because their partner had already participated in an interview. Nineteen parents participated in 18 interviews, as one of the interviews included both parents. In total, 14 mothers and 5 fathers aged between 22 and 35 years were interviewed.

### Data collection

A interview guide was developed, based on the home-visit programme, see supplementary file 1. The first author performed all but one interview, which was performed by the second author. None of the authors had any clinical connections to the participants, but both authors are trained paediatric nurses. All interviews were carried out by telephone and lasted between about 15 and 35 min. The interviews were recorded and transcribed verbatim by an administrator working at the CHS management level, with no contact to the parents. Transcriptions were anonymized and names were removed before analysis.

### Data analysis

A reflexive thematic analysis inspired by Braun and Clarke [33–35] was performed. The interviews were analysed as one dataset. For familiarization with the data, the transcripts were read through by the authors individually. During the reading, brief notes were written in the margins. In the next step, data units were identified and entered into an Excel sheet. The units were then condensed into codes. The initial codes were discussed between the authors and reflected upon in relation to the whole dataset and the study’s aim. Then, the codes were clustered into common themes and initial labels were generated in collaboration, see example Table 1. In the next step, the themes were further developed and refined, referred back and forth between codes and data, and expanded to cover the whole data set in a thoughtful and reflexive immersion to create a common understanding. The broader meanings were interpreted as the themes were further reframed on a conceptual level. Throughout the analysis process, emerging themes were reflected and a core understanding of the data emerged through reflexive elaboration between the two authors on several occasions. The results are presented as analytic narratives [33]. The quotations used to illustrate the results were translated from Swedish by the authors and checked by a qualified language reviewer. The subthemes are illustrated with quotes from the interviews, the interviewees are identified as mother (M) or father (F), followed by the interview number.

**Table 1:** Example of data analysis *

Data unit	Condensed data	Code	Initial theme	Final subtheme
Comparing to visits at the CHS, the child feel more safe at home, in her known environment, then it is easier for the staff to judge the child development as well	The staff will see how the child behave at home	assess behaviour	security at home	*Home environments increase the feeling of security*
Comparing to visits at the CHS, the child feel more safe at home, in her known environment, then it is easier for the staff to judge the child development as well	Child feel safe in known environment	known environment		
We know that there is a limited time for the visits, but it did not feel like that since the staff were so calm and relaxed	The staff seemed more relaxed	relaxed feeling	relaxed environment	
It feels safer talking about sensitive matters at home, at the CHS there are others in the waiting room, and you don’t want to come out there crying	Talking about sensitive matters	sensitive matters	secure feeling	

^*^Transcripts and analysis were in Swedish, and the example has been translated by the authors

### Ethics

The Swedish Ethical Review Authority approved the study, reference number 2021–04925. The study was performed in accordance with the Declaration of Helsinki and Swedish research regulations. All participants got and signed an informed consent form when leaving their phone numbers, and they verbally agreed to participate when the interview was booked and once more when it started.

## Results

**Fig. 2:**
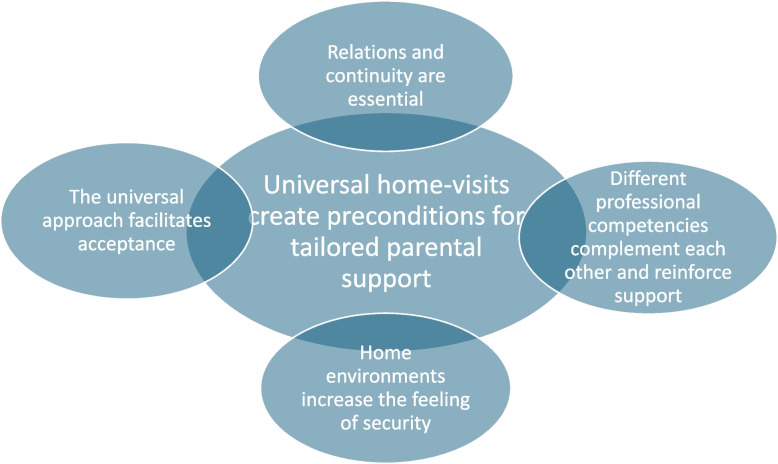
Overarching theme and subthemes

The results consist of one overarching theme, *Universal home-visits create preconditions for tailored parental support*, and four subthemes: *Relations and continuity are essential*; *Different professional competencies complement each other and reinforce support*; *Home environments increase the feeling of security*; and *The universal approach facilitates acceptance* (Fig. 2).

The results illustrate the mainly positive experiences expressed by the parents. Even if they did not always understand the purpose the programme served for their particular family, they thought it was good for all first-time parents and the general approach worked proactively and preventatively, thereby promoting children’s health and development.

### Relations and continuity are essential

The parents expressed the importance of continuity in building trustful relationships with both the CHS nurse and the social worker. This was important in embracing the possibilities of the parental support that was offered.

The first step in building a relationship was to understand the purpose of the extended home-visit programme. This was only sometimes clear to the parents, or at least they expressed that they were not sure. Before the first home-visit, most parents wanted to “clean up” to show a presentable image of their home. However, this was not anything they thought about when the professionals came; no one felt that checking the home was the purpose of the health visit. The parents expressed that providing them with a clear description of the purpose of the home-visit beforehand was something that could be improved in the home-visit programme.

The parents described a relationship that developed over time through the repeated home-visits. This strengthened them in their new role as first-time parents and made them more secure in their parenthood. According to the parents, meeting the same CHS nurse and social worker at all visits facilitated dialogue and made it easier to speak up about more difficult issues.

*“It has felt nice and lovely. We have got to know them a little now, it’s almost like it’s two friends coming home to you. Just having followed each other so closely all the time.”* [M14].

As a first-time parent with a newborn baby, building a relationship and having access to different and complementary competencies was experienced as an opportunity to get support both now and later, if needed. The extended home-visits were also a ground for closer contact with the social worker and not just the CHS nurse, whom the family met for regular health visits at the Family Centre between the home-visits.

The parents expressed that there were no logistic issues; they could change the times for their appointments if needed and adjust the times for a working parent so that both could be present most of the time. The parents described it as an advantage if both could attend the home-visits, and the professionals also encouraged it. At the same time, some highlighted that there also needs to be an option to talk alone. In some cases this was done when the CHS nurse took one parent and the baby to take measurements while the social worker could talk with the other parent.

More hands-on advice based on the home situation and the child’s development was also described as being more accessible when getting several home-visits. The parents expressed that if they forgot to ask something, or when a question arose, if they knew that the professionals would come back soon, or they had a visit at the Family Centre in between, they did not have to call; they saved the questions until the next visit.

*“But some things you might go and ponder, then you kind of think, I don’t need to call and bother [someone] and talk to someone I don’t know, if I know that I’m going to have a visit and that it’s not so urgent. Then I think it’s really good to collect it [the questions].” *[M6]*.*

All participants expressed that relations are important. Meeting the same CHS nurse and social worker at all the home-visits also made it possible for the relationship to progress. They could continue where they left off the last time or even postpone a discussion to the next visit.

### Different professional competencies complement each other and reinforce support

The parents expressed a high degree of trust and confidence in the professionals’ competence, expecting them to detect if anything was not right concerning their child, their home, or their parenthood. They thought the CHS nurse and social worker would detect any factors affecting the child’s health and development during the home-visits. According to the parents’ experiences, the professionals acknowledged the parents and their home as good enough. This implied that the parents felt secure as first-time parents.

When two of them were working together, the parents thought that the professionals’ ability to detect different issues increased and that they also supported each other. The CHS nurse and the social worker complemented each other based on their professions and had a slightly different focus during the home-visits. The social worker contributed to a focus on the whole family’s well-being, not just that of the child, which often was the case when visiting the CHS nurse at the clinic. The parents appreciated this.

The parents described how their main focus was on the child, especially when it was a newborn. Through the dialogues during the home-visits, the professionals’ different perspectives were helpful in their parenthood and helped them to also take their own well-being into account. Especially fathers thought they were more visible when the social workers were present during the home-visits. The social workers had time for them and could discuss issues that related more to the parents and their feelings and relations.

*“But it may be easier for the social worker to see if there is something that is in a bad state or if there is something you need to talk about. Then it felt like the social worker maybe focused a little more on the parents, on mental and physical well-being and the like. And it feels as though you maybe both don’t really have the time and that it may not be what the CHS nurse has as a direct area of expertise.”* [M11].

At the same time, the two professionals had a smooth transition between the different parts of the visits, where their different skills strengthened the parents’ opportunities to receive parental support. It was described as a gift to have the opportunity to be offered support from the social worker at home and be given information about the different possible forms of support they could receive from other parts of the Family Centre, such as parental groups and an open pre-school. The smooth dialogue between the professionals and the parents about different aspects of parenthood shed light on aspects the parents had not thought about earlier.

### Home environments increase the feeling of security

Being at home was practical; the parents said they did not need to go out to the clinic with their child, which perhaps was a longer distance. Staying in their comfort zone at home was also convenient and contributed to the parents’ feeling of security in the encounter with the professionals.

The parents experienced that being in their home contributed to feeling more secure and relaxed. They also thought their child behaved more confidently and more as usual in a well-known environment.

*“Then, more at around 8 months you could see how she was in her safe environment, compared to when we are at the Family Centre, she is a little scared because she doesn’t really know what is happening.”* [M1].

The home-visit was calmer and less stressful than the visits to the Family Centre. According to the parents, being more at ease in their home helped them to listen and take in information better. The parents experienced that they could steer the dialogue better during the home-visits, which was essential for them, even if the CHS nurse had an overarching agenda based on the Swedish national programme of what should be done.

The parents also expressed that the professionals seemed more relaxed and had more time for them at the home-visits than at the Family Centre. The parents understood that the time was limited, but the professionals did not show it and contributed to a relaxed atmosphere.

*“It feels very relaxed when you’re sitting in your own home … you felt seen, that they actually took the time and saw our situation …”* [F9].

Further, the parents described more easily being able to open up and discuss sensitive matters during home-visits than during visits at the Family Centre. Being in their own home made it easier for them to dare to open up and describe difficulties in their new situation. When visiting the Family Centre, it was easier to keep it together, showing a façade to the CHS nurse and others at the Family Centre. If you were emotional, you did not need to meet others when leaving. One mother said:

*“There is no one else there [at home] who can hear me or see me, well, when you go to the Family Centre there can be others of the same age who are there … then you don’t want to walk out of there crying … if there is anything that’s difficult, that you want to talk about or something …”*. [M15].

The parents also expressed that even if the professionals had a to do list for the home-visit, it was easier for the parents to control the conversation at home. It was also calmer at home; sometimes the parents could notice a feeling of stress at the Family Centre, knowing there were others in the waiting room. At home they felt that there was no time limit, even though they knew there was.

### The universal approach facilitates acceptance

Despite some initial thoughts about the purpose, none of the parents questioned or turned down the home-visits; instead, they appreciated it as an appealing offer to all families.

The parents expressed that including all families, not just those that were expected to have problems, made it less problematic for all.

*“Yes, but I think everyone needs it, because it’s very easy to keep up a façade too when you’re at the visits [at the Family Centre]. You clean up a bit and say what you think they want to hear.”* [F9].

All parents had received information before the first visit, but not all had understood the purpose. Still, no one turned them down, which they thought was because CHS is a well-known concept. They also hoped that this approach really could help children and families who had greater needs than them. The parents believed that it would be harder to cover up and hide issues if the professionals made home-visits to all. It could make it easier to identify those who had greater needs and thereby it would, in the long run, benefit children and families that otherwise may not be identified.

Even parents who did not think beforehand that they needed the extended home-visits experienced that they were offered support they probably would not have requested or even known was available otherwise. Being introduced to a social worker already when the baby was a newborn was experienced as facilitating contact later if the need arose.

*” … that you were offered one of those [visits with the social worker] … I think … if there is a need.”* [F16].

There was a larger focus on the whole family and their situation, not only on the child, which often is the case when meeting the CHS nurse at the Family Centre. It was also easier to take part in other activities at the Family Centre, such as the open pre-school, when having received more information at the home-visit.

The parents said that some probably never would contact a social worker otherwise, but this made it less scary or strange. An example was that the social worker was someone you only had contact with when there already was a big problem. This approach – meeting a social worker when the baby was a newborn – made it more normal to talk to a social worker as part of parental support at the Family Centre.

## Discussion

Becoming a parent is a large transition in life, and to improve care, the Region adopted and introduced an extended home-visit programme [25]. The programme has developed out of an agreement between the state and the health care regions in Sweden [30]. The results in this study show that the extended universal home-visits create preconditions for more family-tailored support and strengthen the first-time parents in developing their parenthood. The discussion is framed around the microsystem model, somewhat adjusted from the CHS perspective (Fig. 1).

### Society perspective

From an overall society perspective, the participants in this study thought that extended home-visits could benefit society by making it possible for the professionals to detect needs earlier. The parents said that the purpose of the extended home-visit programme could have been made clearer, as they did not always remember getting that information beforehand Britto et al. [15].

According to the Convention on the Rights of the Child [39], all children should be able to have a good healthy childhood. CHS in Sweden is a universal offer to all families and is used by almost all [40]. However, usage has been lower in some socioeconomically vulnerable areas, and the Child Health Service Accessibility Agreement [30] was established for this reason. Government health care reforms can function as a steering mechanism [41] and this agreement is seen as an initiative to reduce inequity. In the Rinkeby area, the home-visit programme has shown gaining understanding of the society overall and particularly available services for parents [36, 42]. However, what is expected of authorities from a society perspective does not always correspond to what municipalities from a community perspective are able to accomplish. There can be challenges in connecting special funds to the day-to-day work, and issues such as timing and continuation, prioritization, and funding stability affect the local organization and their decisions [43]. The extended home-visit programme was carried out in a whole municipality, not only in socioeconomically vulnerable areas, which differs from other projects (e.g. [16, 19, 37]). Therefore, our results give a broader perspective of such reforms.

If the parents had any initial fears of having a social worker come home to them, these fears disappeared after the first home-visit. It was experienced as a benefit and as an opportunity to build a closer relation. Other studies show that relations towards the authorities in Sweden in general are trustful [2, 13, 20]. The parents expressed that it was an advantage that, with a few exceptions, the same CHS nurse and social worker came to all home-visits, which helped in building trust. That trust can be created through multiple visits corresponds to findings from previous research [25, 44].

### Community perspective

Based on the results from the Rinkeby programme [16], several regions in Sweden have expanded the universal national programme, adding several home-visits by social workers and CHS nurses to families mostly in more socioeconomically challenging areas [17–19, 31].

The present programme was first piloted in one of the region’s municipalities. Although this municipality was chosen due to its socioeconomic challenges, the region decided, in cooperation with the municipality, to address all first-time parents in the area. The Swedish CHS is built upon proportional universalism [12], and the region and the municipality wanted this initiative to also be a part of it. Avoiding stigmatization can be important when building trust in the community and authorities [44]. The Family Centre was already established and CHS and social care were located in the same place, which was experienced as a precondition for making this extended home-visit programme work [25]. The parents in this study did not express that they had noticed any logistic problems. It was also easier to use other facilities at the Family Centre when introduced by the CHS nurse and social worker at the extended home-visits.

### Family Centre perspective

The fathers expressed feeling more involved when there were two professionals, with the CHS nurse mainly concentrating on the child and the mother while the social worker could be there for the fathers. In a study by Tiitinen et al. [42] the fathers expressed that the home-visits strengthened their confidence as a first-time parent. This finding differs from Solberg et al. [21], where the fathers felt they were not included enough as first-time parents with insecurities and needs. It is important for the CHS to pay attention to both parents, and the extended home-visits can contribute to improving the family-centred perspective [22, 24]. Collaboration between different professions, here CHS nurses and social workers, can improve the support provided [25]. It is important to bear in mind that no two families are the same, and the purpose of a family-centred approach is to recognize not only the family as a whole but also the individuals in the family and their specific needs [24]. The parents, and especially the fathers in our study, expressed they felt more seen by the social worker; not that the CHS nurses did not see them, but they focused mostly on the child after all. Therefore, the CHS nurse and social worker complemented each other.

The parents noted a difference in how the professionals acted when at their home compared to the Family Centre, perceiving them to be more relaxed and attentive during the home-visits. It was more stressful at the Family Centre, knowing others were sitting in the waiting room. Although the parents understood that the professionals did not have an unlimited amount of time, it felt as though they did during the home-visits. Such a relaxed feeling can underpin a good relationship [20, 21].

Working at a Family Centre requires teamwork and collaboration [45]. The parents thought that the professionals complemented each other. Meeting two different professions, with slightly different approaches and focuses, improved the extended home-visits. It also made them more valuable than the visits to the CHS where the social workers seldom participated. In the study by Golsäter and Andersson [25], the CHS nurses and social workers highlighted that collaboration was key; the different professions and competences completed each other and increased the potential to add value for the children and families.

### Family perspective

Becoming a parent for the first time is a transition in life [8, 32] that sometimes can be overwhelming. Extended home-visit programmes are one effort to support new families. Many families today do not have their own parents close by, which makes the CHS and Family Centre the main support in the new situation [7]. The professionals collaborating with the whole family were found to be an advantage, which the professionals themselves also thought was important [25].

A review identified different models for family-centred care, of which most were designed for paediatric populations and different illnesses [23]. The purpose of extended home-visits is to support families and detect any issues early to avoid problems [25], which the families in this study appreciated. Research has shown that first-time parents need support from family as well as social and health services in the transition to becoming a parent [6]. Nurturing care is seen as essential to promote a safe childhood and fulfil the child’s developmental potential [2, 3, 15]. Therefore, professionals working in CHS need to provide tailored support [46]. The extended home-visits in a secure home environment can support and encourage first-time parents when becoming a parent. Just like the professionals in an earlier study [25], the participants in this study emphasized the usefulness of the programme at a microsystem level, making it beneficial to the whole family.

#### Strengths and limitations

First-time parents who had taken part in the extended home-visit programme were informed about the study by the CHS nurse. Those who were willing to participate in the study gave their consent and their phone number to be contacted by the researchers. We do not know how many parents were asked and how many refused; it could be that those who were positive chose to participate. However, the data gathered was rich and described the parents’ various experiences and thoughts about the home-visits. In thematic analysis, data richness and information power are important [24, 33]. Quotes from the parents’ descriptions were used to illuminate the content of the themes and as a way to strengthen trustworthiness.

Subjectivity is always an issue in qualitative research and needs to be considered. In the reflexive approach, researchers take an active role in the construction of knowledge and the interpretation of data, but at the same time they need to be aware of their assumptions and positioning to enable credibility and trustworthiness [33]. The reflexive approach accentuate the researcher’s role in knowledge production [34]. Both authors are trained as paediatric nurses, which influence the reflexive part of the analysis and conceptualisation of data. They also have extensive experience of conducting qualitative studies and interviews as well as performing reflexive thematic analyses. The authors actively collaborated in the analysis process, which contributes to a richer and more nuanced reading of the data that goes beyond simply seeking consensus. Thematic analysis allows more multi-dimensional themes, which are readily accessible and contribute to broader confirmability and transferability [34, 35].

## Conclusions

The study shows that the interviewed first-time parents appreciate the extended home-visits. The extended home-visit programme can be applied to a whole municipality and gain all first-time parents, not just those in socioeconomically vulnerable areas. Building trust with both a CHS nurse and a social worker can contribute to parents getting a positive image and confidence in what the Family Centre can offer. Even parents without any major problems were strengthened in their parenthood. They perceived that the model provided a way to initiate recurring opportunities for dialogue about the family’s situation and to receive support if needed. The extended home-visit programme encourages families to have easier access to other activities and support functions within the Family Centre’s commission and supply.

Based on the parent's perspective, the results from the present study can be used on a community level when introducing a home-visit programme in municipalities where areas of socioeconomic vulnerability are more scattered. The results show that all parents expressed benefit from the programme, and by that, also the concerns about feeling singled out or stigmatized were solved.

From an international perspective, CHS nurses can use these results to elaborate their work;

- At a family level, the results illuminate the opportunity to build trustful, family centred caring relationships through home-visits compared to clinic visits.
- At the Family Centre level, the parents experienced that the home-visits created more opportunities to meet with the social worker on a more regular basis and to have access to the Family Centres as a whole. These results can be used when developing Family Centres in Sweden and internationally, e.g., in the Nordic countries having an organisation of CHS similar to the organisation in Sweden.

Further research to study the outcome of home-visit programmes on children’s health and development would be helpful. In addition, research regarding how other parts of a Family Centre, the midwives and the preschool teachers, could contribute to the home-visit programme would also be interesting.

## Supplementary Information

Supplementary Material 1.

Supplementary Material 2.

## Data Availability

All data generated or analysed for this study are included in this published article. The anonymized dataset can be made available by the corresponding author upon reasonable request.

## Abbreviations

CHS: Child Health Services
CHS nurse: Child Health Services nurse
