# Supplementary material for: A universal home-visit programme to tailor support to first-time parents: a qualitative case study on parents’ perspectives
Source: BMC Public Health. 2025 Sep 9;25:3045. doi: 10.1186/s12889-025-24114-z (PMC12418607; doi:10.1186/s12889-025-24114-z)
Supplement: Supplementary file 1 — Supplementary Material 1. [file 12889_2025_24114_MOESM1_ESM.docx]

**Reflexive Thematic Analysis Reporting Guidelines (RTARG)** by Virginia Braun & Victoria Clarke (2024). A list of advice for aspects of the research report/approach.

| **Topic** | \| **Advice for aspects of the research report/approach to reporting** \| \| --- \| | **Reported in Page No.** | **Comments ^a^** |
| --- | --- | --- | --- | --- |
| **The Introduction** | | | |
|  | We prefer *Introduction* over *Literature Review* as a section heading, to capture the broader purpose of this section. |  | We have used headings corresponding to the journal author guidelines. |
| ***Background and rationale*** | | | |
| \|  \| \| --- \| | Provide a robust context and rationale for the proposed research in the *Introduction.* | Pages 4, 5 |  |
| \|  \| \| --- \| | Clearly articulate a research question – one that is methodologically coherent. | Page 8 | We use an aim for this study in line with the journal author guidelines |
| ***Owing your perspectives*** | | | |
| \| \|  \| \| --- \| \| \| --- \| --- \| | Include information on guiding theoretical assumptions and other (e.g., explanatory) theory informing the use of TA. | Pages 6-7 | The microsystem theory is the foundation of this work, both the extended home-visit programme and the study is based in the context of the Region QI approach. |
|  | Report in a way that is consistent with stated theoretical assumptions throughout. | Result section, pages 12-18 |  |
|  | Evidence methodological coherence/integrity in both the research and the report. | Method section, pages 10-11 |  |
| . | Show evidence of reflexive practice. | Pages 10-11 |  |
|  | Write in a methodologically coherent style. | Method section, p. 9-11 |  |
| **The Methodology** | | | |
|  | We prefer the theoretically-embedded term *Methodology* as a section header, over the proceduralist term *Method.* |  | We have used headings corresponding to the journal author guidelines. ^a^ |
| ***Participants/data items*** | | | |
|  | Describe selection of participants/data items. | Page 10 |  |
|  | Describe number of participants/data items; provide a rationale or explanation around dataset or participant group size/composition. | Page 10-11 |  |
|  | Discuss characteristics of participants/data items. | Pages 10, and strengths and limitations, p. 23-24 |  |
|  | Detail ethical approval and ethical code/principles followed, participant informed consent, etc. | Page 11-12 |  |
| ***Data set generation*** | | | |
|  | We prefer the term *generation* over *collection* to capture the active role of the researcher and that data don’t pre-exist research *as* data, but *become* data through research practices. |  | We have used headings corresponding to the journal author guidelines. ^a^ |
|  | Provide some rationale for method(s) for data generation/data item sources chosen. | Pages 9-11 |  |
|  | Describe development and/or characteristics of data generation tool(s). | Page 11 |  |
|  | Include details such as modality and/or setting of data generation, time frame, and other pertinent procedural information. | Page 9 |  |
|  | Describe who conducted any interactive data generation (which author or research role), and how. | Pages 10-11 |  |
|  | Describe the size/scope of dataset and dataset items. | Pages 9-11 |  |
|  | Describe, and if relevant explain, any preparation of data for analysis. | n.a. | No special preparations were made. |
| ***Data analysis*** | | | |
|  | Provide some rationale for use of RTA, and, where relevant, for combining RTA with other approaches and procedures. | Pages 10-11. |  |
|  | Describe specific orientation to RTA. | Pages 9-11 |  |
|  | Discuss how the researcher(s) engaged with the analytic process. | Pages 10-11 | We have added some text about the researcher’s preunderstanding as trained paediatric nurses, which influence the reflexive process. |
|  | Where more than one person is involved, describe who analysed the data (author or research role). | Pages 10-11 |  |
|  | Use language to describe the process and products of RTA that is coherent with the values and assumptions of RTA. |  | We have used the language of the author guidelines. |
| **The Analysis** | | | |
|  | We prefer the heading *Analysis* over *Findings*/*Results*. *Findings* implies the researcher “found”, “discovered” or “identified” pre-existing themes. *Results* is strongly associated with the outputs of statistical analysis. |  | We have used headings corresponding to the journal author guidelines. ^a^ |
| ***Reporting the data analysis*** | | | |
|  | Provide an overview of themes or thematic structure. | Page 12 and fig. 2 |  |
|  | Ensure theme conceptualisation is appropriate to RTA, and any divergences are justified and explained. | Method section |  |
|  | Name themes appropriately. | Page 12 |  |
|  | Report themes in sufficient depth and detail. | Result section |  |
|  | Use subtheme judiciously. | Result section |  |
|  | Ensure the analytic narrative explains the meaning and significance of the data. | Result section |  |
|  | Provide an appropriate *balance* of analytic narrative and data extracts – both data extracts *and* analytic narrative matter. | Result section |  |
|  | Demonstrate coherence between analytic narrative and illustrative/evidentiary data extracts. | E.g., citations in result section |  |
|  | Integrate existing research and theory into the analytic narrative. | Discussion section |  |
| **The Final Section – A General Discussion or “Conclusions”** | | | |
|  | We don’t have a preference for what a final section of an RTA report is called, and it depends on the context and the focus and purpose of the study – the heading *Conclusion* may evoke a certainty that isn’t appropriate; *Implications* may be useful; *Final Considerations* or *Reflections* may work, as might *General Discussion.* |  | We have used headings corresponding to the journal author guidelines. ^a^ |
| ***Quality, evaluation and conclusions*** | | | |
|  | Draw analytic conclusions across themes. | Discussion section |  |
|  | Discuss implications or directions for future research. | Conclusion section page 24-25 |  |
|  | Use and report quality practices coherent with RTA. | Strengths and limitation section, p. 23-24 |  |
|  | Evaluate the research from a *Big Q* standpoint. | Conclusion section, p. 24-25 | As a first municipality in the region starting this concept of Extended home-visits, it will inform the introduction in the rest of the region, and as including all first-time parent, not only socioeconomical vulnerable areas, the results can inform a broader audience as well. |
|  | Include reflections on research process and practices, including researcher reflexivity. | Strengths and limitation section, page 23-24 |  |

^a^ When listed advises are incoherent with praxis in the journal, the praxis of the journal is followed.
