# Supplementary material for: A universal home-visit programme to tailor support to first-time parents: a qualitative case study on parents’ perspectives
Source: BMC Public Health. 2025 Sep 9;25:3045. doi: 10.1186/s12889-025-24114-z (PMC12418607; doi:10.1186/s12889-025-24114-z)
Supplement: Supplementary file 2 — Supplementary Material 2. [file 12889_2025_24114_MOESM2_ESM.docx]

**Interview guide:
Parents' experiences of extended home-visits**

**Introduction**

At at Child Health Care, a project with extended home-visits, where new families are offered joint home-visits by a CHS nurse and a social worker, has been introduced.

Evaluating new work methods is an important part of this process, and this study examines parents' thoughts about this method.

**Background information:**

Mother, father, other

Age

Experience of the extended home-visit programme

Number of joint home-visits

What do you think about families being offered joint home-visits by a CHS nurse and a social worker?

- How were you informed about the joint home-visits, that a CHS nurse and a social worker would come to you together for a home-visit?
- What was it like to receive a joint home-visit?
- Did you get the opportunity to discuss what you wanted with the child health nurse and the social worker?
- How did you experience the CHS nurse and the social worker's competence talking to you?
- Were you offered more/other support based on what emerged during the home-visit?
- If yes, what do you think of the support that was offered?

What is the difference between home visits and CHS/Family Centre visits?

- What aspects do you think are most important to talk about during home-visits?
- What could improve the design of home-visits?
- What is essential to include in the continued development of the work with the extended home-visits programme?

Is there anything else you would like to tell us?
